# Functional Outcomes of Robotic-Assisted Total Knee Arthroplasty in Severe Varus Deformities of the Knee in the Indian Population

**DOI:** 10.7759/cureus.72398

**Published:** 2024-10-25

**Authors:** Jagadeesh P Chamundaiah, Nirav R Gupta, Senthilkumar Mahalingam, John Edwin

**Affiliations:** 1 Department of Orthopaedics, Kauvery Hospital, Electronic City, Bengaluru, IND; 2 Department of Orthopaedics, Royal Cornwall Hospital, Truro, GBR; 3 Department of Orthopaedics, Basildon University Hospital, Basildon, GBR

**Keywords:** clinical outcomes in severe deformities, functional alignment, functional outcomes, indian population, robotic-assisted total knee arthroplasty, severe deformity, severe varus

## Abstract

Purpose: Correction of severe coronal plane deformities while performing a total knee arthroplasty (TKA) is challenging. The use of functional alignment (FA) strategy along with image-based robotic technology during TKA makes it possible to restore a patient’s constitutional alignment with minimal or no soft tissue release. The present study aims to report the functional outcomes of robotic-assisted TKA in Indian patients with severe varus deformity.

Methods: This retrospective study included 82 primary TKA performed by a single senior arthroplasty surgeon from January 2022 to December 2022. The Visual Analogue Score for Pain (VAS-P) and the Knee Injury and Osteoarthritis Outcome Score for Joint Replacement (KOOS-JR) were used to assess functional outcomes. A comparison between mild and severe varus deformities was done using Student’s t-test.

Results: The mean age was 66.35 ± 9.21 years. There were 79 (96.34%) knees with a mean preoperative varus hip-knee-ankle angle (HKA) of 12.85° ± 6.03° (range = 177°-148°). Postoperative limb alignment was achieved in the range of HKA 174° to 180°, with mean HKA being 175.78° ± 1.35°. The VAS-P improved significantly from a mean of 7.87 ± 0.81 preoperatively to 1.96 ± 1.43 at 18 months. KOOS-JR improved from 41.22 ± 7.6 preoperatively to 88.44 ± 8.23 at 18 months. A total of 73 (89.02%) cases achieved the minimal clinically important difference (MCID) for VAS-P and 77 (93.9%) cases achieved the MCID for KOOS-JR in our study. Functional outcomes were comparable among the mild (<10°) and severe (>10°) varus deformity cases at 18 months follow-up (p-value > 0.05). There was a significant difference in bone resection from the medial tibial and medial distal femoral condyle (p-value < 0.05) with less bone resected in the severe varus group. The amount of bone resected from the lateral tibial, distal lateral femoral, and posterior femoral condyles was comparable among the two groups (p-value > 0.05). FA was more bone conserving even in severe varus-deformed knees when compared to mechanical alignment (MA).

Conclusion: The use of FA with robotic assistance to correct severe varus deformities in TKA showed a significant reduction in VAS-P scores and an improvement in KOOS-JR scores. The functional outcomes were comparable with TKA in mild varus deformities. FA helps in preserving bone from the lateral tibial condyle and distal femur.

## Introduction

Correction of severe coronal plane deformities while performing a total knee arthroplasty (TKA) is challenging. Residual malalignment can be detrimental. Traditional teaching for a successful TKA comprised aligning the knee neutral to the mechanical axis (MA) of the lower limb. This was to provide successful outcomes with excellent implant survivorship. However, 20% of patients were still dissatisfied and never had a feeling of natural joint post-TKA [1,2]. With the TKA implants still in infancy in the early 90s, the main focus was on implant survival. Now with a more detailed understanding of knee biomechanics and excellence in metallurgy, the aim of a TKA is to restore near-natural knee function [3]. The kinematic alignment (KA) was introduced to achieve this. A simulation study by Ishikawa et al. [4] compared the mechanically aligned knees with kinematically aligned knees on a single knee model and concluded that the KA TKA produces near-normal knee biomechanics.

A hip-knee-ankle angle (HKA) between 170° and 177° is considered a mild varus and less than 170° is considered a severe varus [5,6]. Similarly, HKA beyond 200° is severe valgus [7]. In kinematically aligned TKA with severe deformities, there were increased contact stresses on the polyethylene and tibial base plate, which raised concerns for implant failure. In recent times, with the widespread use of robotic assistance to perform TKA, functional alignment (FA) has become popular. FA is a school of thought that employs the use of image-based robotic technology to TKA to restore a patient’s constitutional alignment, joint line height, and obliquity within set limits by readjusting the implant position based on soft-tissue laxity [8]. It is indeed a true knee resurfacing with minimal or no soft tissue release (STR). In their randomized control trial, Masilamani et al. [9] reported a higher incidence of balanced knee with a significantly reduced need for STR when using FA strategy. It is possible for the surgeons to individualize the preoperative plan and precisely execute it while performing TKA. In their study, Jeffrey et al. [10] reported that FA gives a better clinical outcome in short-term follow-up when compared to MA. However, the maximum preoperative varus deformity in their study was 15°.

There are very few studies in the literature that have evaluated the results of FA in severe varus, none in the Indian population. The present study aims to report the functional outcomes of TKA in Indian patients with severe varus deformity operated using FA strategy with robotic assistance.

## Materials and methods

**Figure 1:**
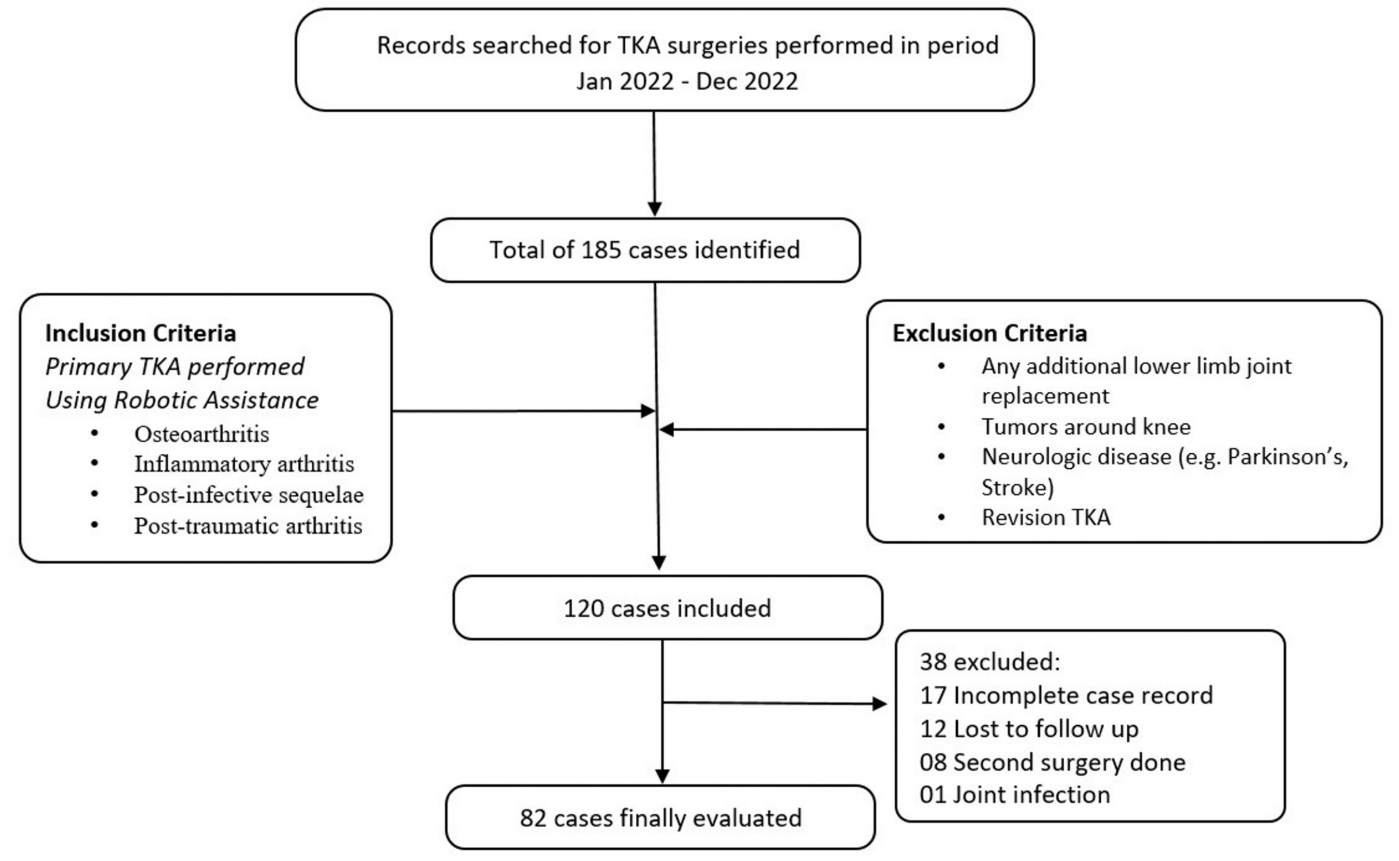
A flowchart depicting patient inclusion and exclusion criteria. TKA: total knee arthroplasty.

TKA surgeries performed from January 2022 to December 2022 by a single senior arthroplasty surgeon in a tertiary care center using image-based robotic assistance (Semi-automated Stryker MAKO Robotic System, Stryker, Portage, MI) were included. Patient’s preoperative, intraoperative, and follow-up data were retrospectively collated and the preoperative deformity, i.e., HKA, intraoperative implant positioning, size and amount of bone resection, postoperative limb alignment (LA), 10 cm Visual Analogue Score for Pain (VAS-P), and the Knee Injury and Osteoarthritis Outcome Score for Joint Replacement (KOOS-JR) were noted from the records. Cases were divided into two groups based on preoperative deformity: mild varus (<10⁰) and severe varus (>10⁰). This retrospective study included patients who had undergone primary TKA for knee arthritis (osteoarthritis, inflammatory arthritis, post-infective sequelae, and post-traumatic arthritis). Patients with incomplete records (17), who required second surgeries (8), who had an underlying joint infection (1), and who were lost to follow-up (12) were excluded from the study (Figure 1).

A total of 82 knees were finally analyzed to compare the VAS-P and KOOS-JR scores between the two groups. Additionally, the minimum clinically important difference (MCID) was calculated for both VAS-P and KOOS-JR scores as a statistically significant difference may not always demonstrate clinical usefulness. The minimum follow-up period in our study was 18 months.

Surgical procedure

**Figure 2:**
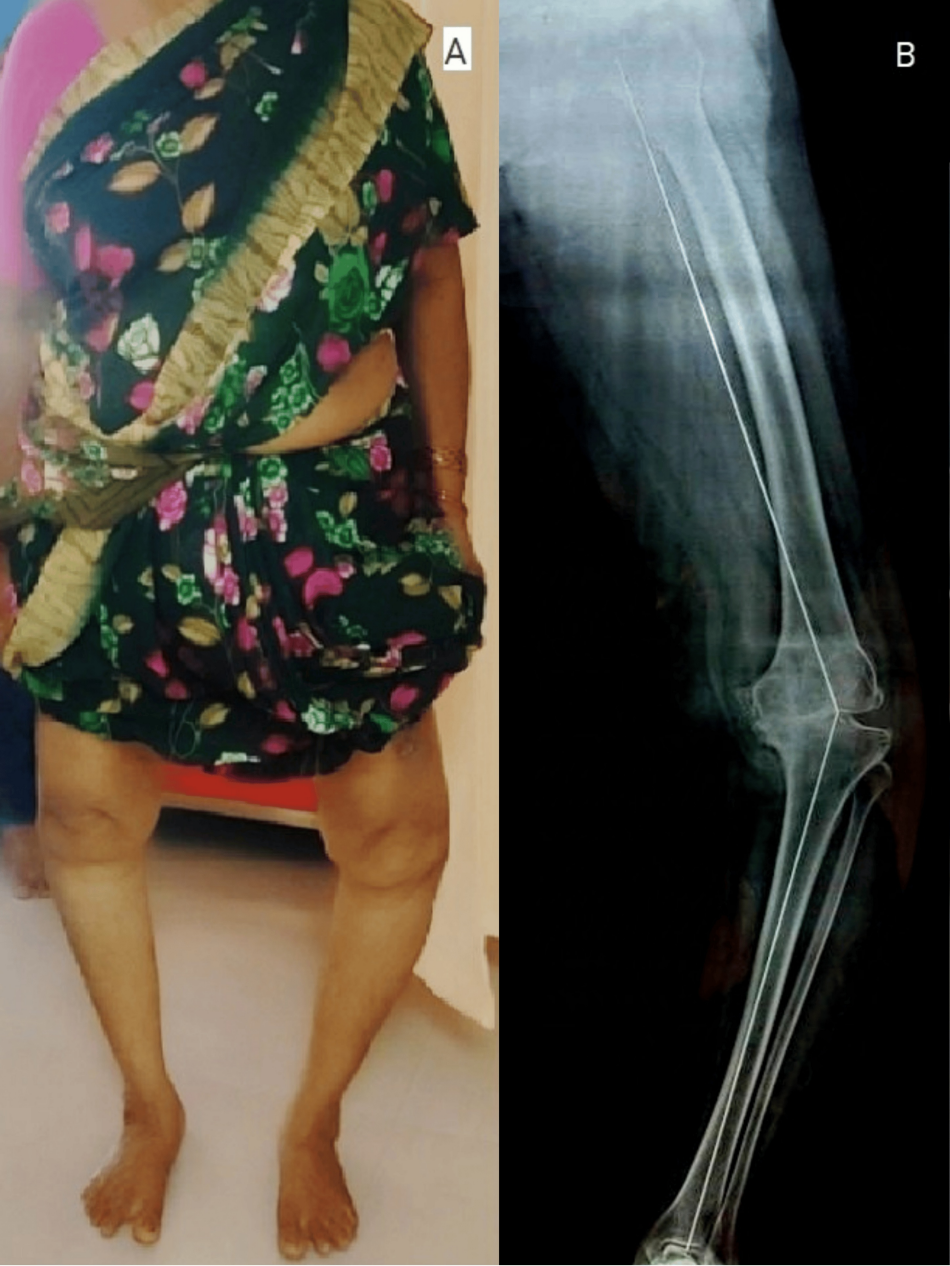
Preoperative clinical image (A) and full leg weight-bearing radiograph of the left lower limb (B) showing severe knee varus deformity.

**Figure 3:**
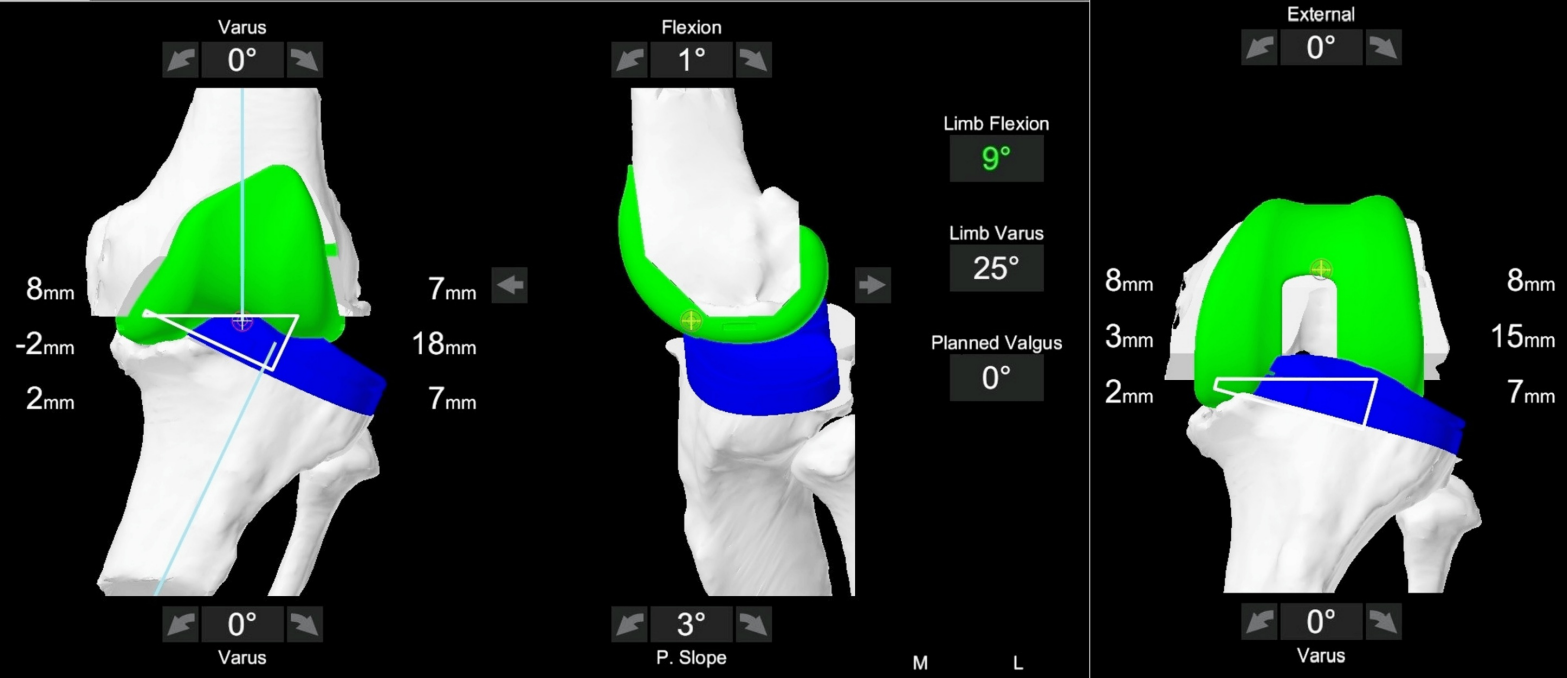
Screenshot from the MAKO robotic software screen after registration at the inception of surgery. Left is the coronal view in extension, middle is the sagittal view in extension, and right is the coronal view in flexion. Varus of 25° has been recorded. The femoral implant is seen in green color and the tibial in blue color. It shows unbalanced extension (-2 mm medially and 18 mm laterally) and flexion (3 mm medially and 15 mm laterally) gaps.

**Figure 4:**
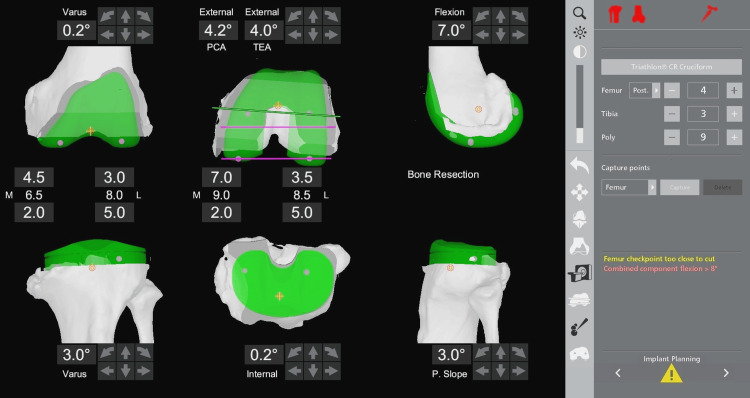
Screenshot from the MAKO robotic software screen at implant planning. The left image is the coronal view, the middle is the axial view, and the right is the sagittal view. Cruciate-retaining implants are seen in green. The femoral component has been placed in 7° flexion and 4° external rotation with respect to the transepicondylar axis. The tibial component is in a 3° varus and 3° posterior slope. Two mm bone is resected from the medial tibial condyle and 5 mm from the lateral. A polyethylene insert of 9 mm is used for planning.

**Figure 5:**
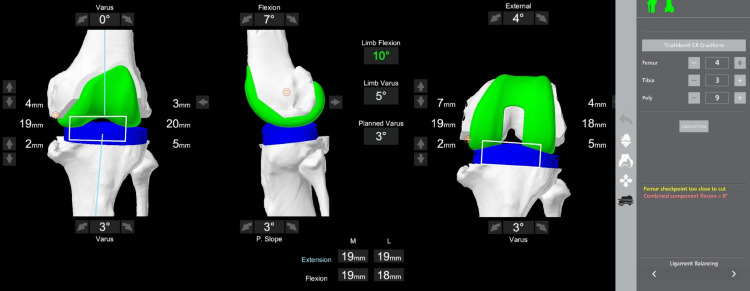
Screenshot from the MAKO robotic software at final ligament balancing. The left image is the coronal view in extension, the middle is the sagittal view in extension, and the right is the coronal view in flexion. The femoral component is depicted by green color and the tibial component by blue color. A very well-balanced extension (19 mm both medial and lateral) and flexion (19 mm medial and 18 mm lateral) gaps can be seen. A limb varus of 5° has been achieved.

**Figure 6:**
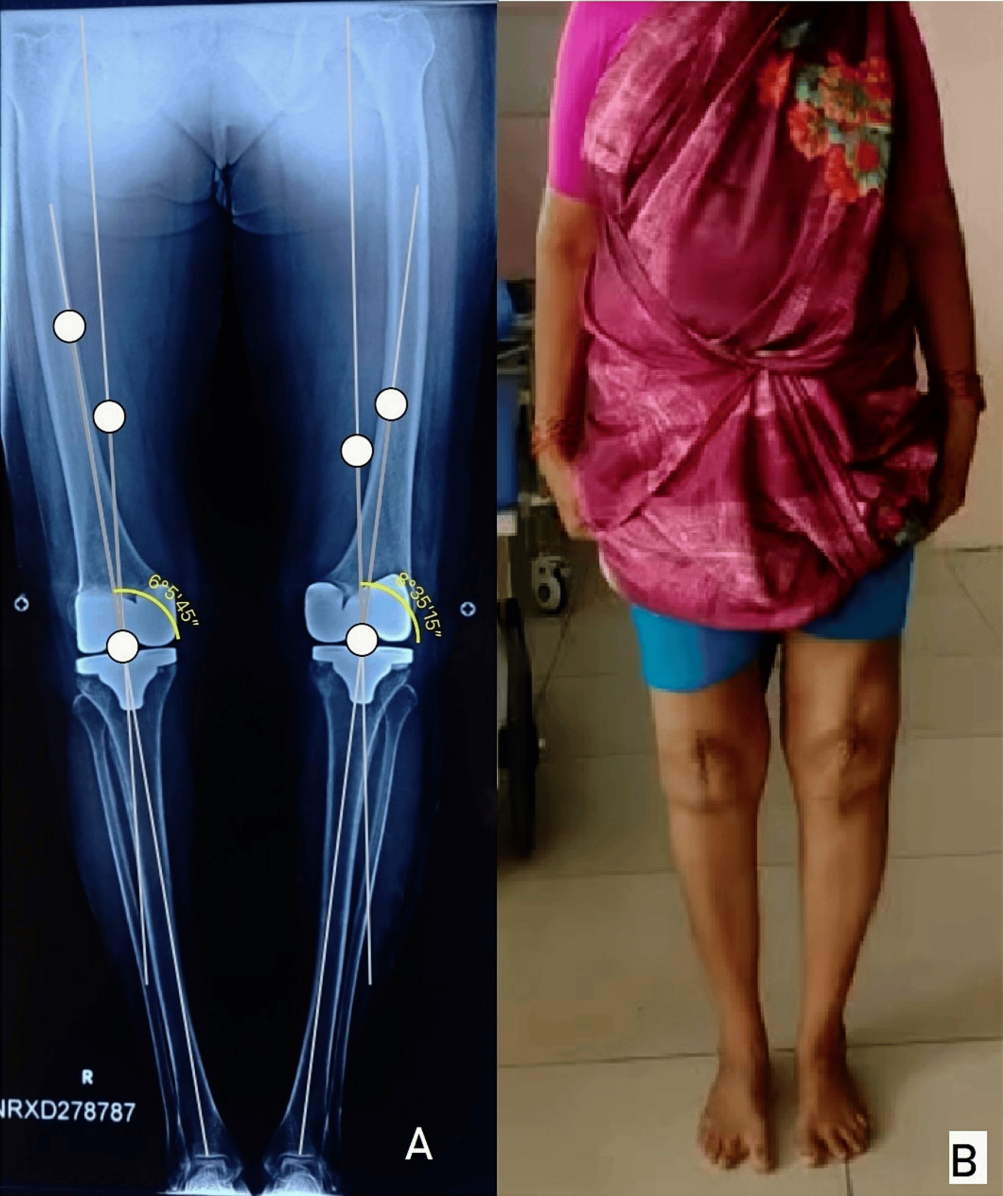
Postoperative full-length standing radiograph of bilateral lower limb (A) and clinical image (B) showing correction of deformity.

A computed tomography (CT) scan of the lower limb with an alignment rod was taken, the data were incorporated into the MAKO robotic software, and preoperative planning was done. As per standard TKA protocol, a midline vertical skin incision was used. Arthrotomy was done using a medial parapatellar approach. The meniscus, anterior cruciate ligament, and overhanging osteophytes were removed. Femoral and tibial checkpoints were placed and after bony registration, the knee was taken through the range of motion in neutral, with maximum possible varus and maximum possible valgus force to record preoperative alignment and gaps. The robotic ligament balancing interface was used to adjust the implant position and size to achieve a well-balanced flexion-extension gap using FA workflow. Once satisfied with the position and overall alignment, the robotic arm was used to take precise femoral and tibial cuts. Minimal deep medial collateral ligament (dMCL) release was done in very severe varus deformities if necessary to achieve balance. With trial implants in situ, the range and coronal alignment were rechecked and recorded. The final prosthesis, Stryker Triathlon - cruciate-retaining (CR) implant, was then cemented in using Simplex® P. Five cases had severe posterior cruciate ligament (PCL) tightness, and therefore a cruciate-substituting (CS) implant was used after performing incremental PCL recession in these cases. Constrained implants were not used in any case. Circumferential patellar denervation and patelloplasty were performed in all cases and patella resurfacing was not done in any of the cases. Closure was done in layers after releasing the tourniquet and achieving hemostasis. No drain was used. IV antibiotic was given 30 minutes before inflating the tourniquet and then for two days postoperatively. Postoperative rehabilitation was started on the same day by allowing in-bed exercises and knee bending. Full weight-bearing walking was started on day one and stair climbing on day two or three, as tolerated by the patients. A representative case with images has been illustrated for the left knee (Figures 2-6).

Statistical analysis

JASP version 0.18.2.0 (The JASP Team, Amsterdam, The Netherlands) and Microsoft Excel version 2013 (Microsoft Corporation, Redmond, WA) were used for statistical analysis. Percentage, mean, standard deviation, and range were calculated for all patients for descriptive data. Functional outcomes, depicted by VAS-P and KOOS-JR score, were reported as mean ± SD at preoperative, 12 weeks postoperative, 12 months, and 18 months follow-up. Differences in functional outcomes among the mild and severe varus deformity were analyzed with Student’s t-test. A p-value of <0.05 was considered statistically significant.

## Results

A total of 47 patients (82 knees) with knee arthritis were included in this study. The mean age was 66.35 ± 9.21 years (range = 51-92 years). Out of these, 12 patients (25.53%) had undergone unilateral TKA, and 35 (74.47%) had bilateral TKA in a single stage. The descriptive data are summarized in Table 1.

**Table 1:** Descriptive data of our study. SD: standard deviation; DOS: duration of stay.

Variable	
Total knees, n	82 (47 patients)
Mean age, years (range, SD)	66.35 (51-92, 9.21)
Sex, n (%)	
Females	58 (70.73)
Males	24 (29.27)
Surgery, n (%)	
Unilateral	12 (14.63)
Bilateral	70 (85.37)
Side, n (%)	
Right	43 (52.44)
Left	39 (47.56)
Mean DOS, days (range, SD)	4.02 (2-7, 1.36)

A total of 79 (96.34%) knees had a preoperative varus LA with a mean varus of 12.85° ± 6.03° (range = 3° to 32°). Out of these, 26 (32.91%) could be classified as grade 1, i.e., varus less than 10°, 44 knees (55.67%) were grade 2, i.e., 10°-20° of varus, and nine (11.39%) were grade 3, i.e., more than 20° varus. Grades 2 and 3 are grouped as severe varus deformity. The mean varus in the mild deformity group was 6.96° ± 2.03° (range = 3°-9°) and in the severe deformity group was 15.74° ± 5.17° (range = 10°-32°). Three knees (3.66%) had a preoperative valgus LA with a mean of 6.67° ± 3.51° (range = 3°-10⁰). One patient (1.22%) had a windswept deformity. One patient (1.22%) had an extra-articular deformity in the distal tibia while all the rest had intra-articular deformities. Preoperative limb deformity is tabulated in Table 2 in detail.

**Table 2:** Preoperative limb deformity in our study. HKA: hip-knee-ankle angle; SD: standard deviation.

Coronal alignment	
Varus, n (%)	79 (96.34)
Mean, degrees (mean HKA, range, SD)	12.85° (167.15°, 177°-148°, 6.03°)
Varus grading	
Grade 1, n (%, mean HKA, SD)	26 (32.91, 173.04°, 2.03°)
Grade 2, n (%, mean HKA, SD)	44 (55.67, 166.16°, 3.01°)
Grade 3, n (%, mean HKA, SD)	9 (11.39, 155°, 2.96°)
Valgus, n (%)	3 (3.66)
Mean, degrees (mean HKA, range, SD)	6.67° (186.67°, 183-190°, 3.51°)
Sagittal alignment	
Flexion deformity, n (%)	69 (84.15)
Mean, degrees (range, SD)	8.08° (1°-16°, 3.92°)
Neutral, n (%)	2 (2.43)
Hyperextension, n (%)	11 (13.41)
Mean, degrees (range, SD)	-3.82° (-1° to -8°, 2.04°)

A postoperative LA was achieved in the range of 0° to 6° varus (HKA = 174° to 180°), having a mean varus of 4.22° ± 1.35° (HKA of 175.78°) in patients with preoperative varus knees. In those with preoperative valgus, the LA was corrected to a mean of 2° ± 1.73° valgus (range = 1° to 4⁰). Hyperextension was completely corrected to neutral and the mean postoperatively attained knee extension lag was 0.34° ± 0.83° (range = 0° to 4°).

Functional outcome was assessed using VAS-P and KOOS-JR scores. The MCID was calculated for both of these based on previously reported MCID in literature. MCID is the minimum amount of change that is considered to be significant. As per the previously reported values, the MCID for the KOOS-JR was calculated to be 15 points, using the anchor-based method [11]. Similarly, the MCID for VAS-P post-TKA was determined to be 2.26 cm for improvement and 2.91 cm for deterioration [12]. A total of 73 knees (89.02%) of cases achieved the MCID for VAS-P and 77 knees (93.9%) achieved the MCID for KOOS-JR in our study.

**Figure 7:**
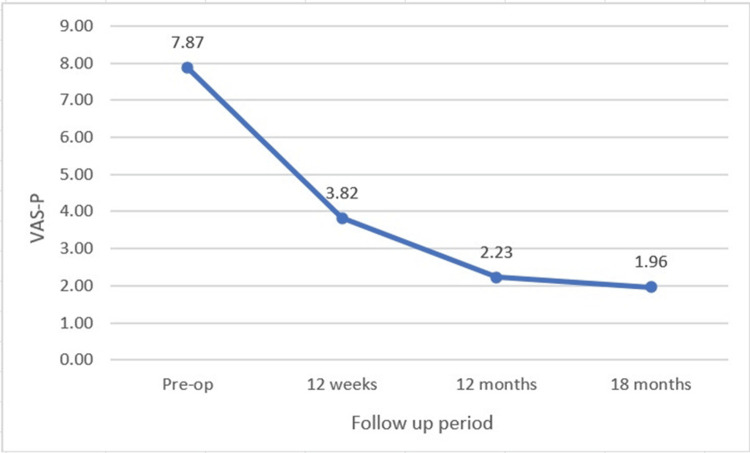
Line diagram showing a decreasing trend of VAS-P score from preoperative to 18 months postoperative period, VAS-P: Visual Analogue Score for Pain.

A significant improvement was observed in these scores at a follow-up of 18 months. The VAS-P score improved from a mean of 7.87 ± 0.81 preoperatively to 3.82 ± 1.18 at 12 weeks postoperatively and to 2.23 ± 1.41 at 12 months and to 1.96 ± 1.43 at 18 months. This change was statistically significant with p-value <0.05 (Figure 7).

**Figure 8:**
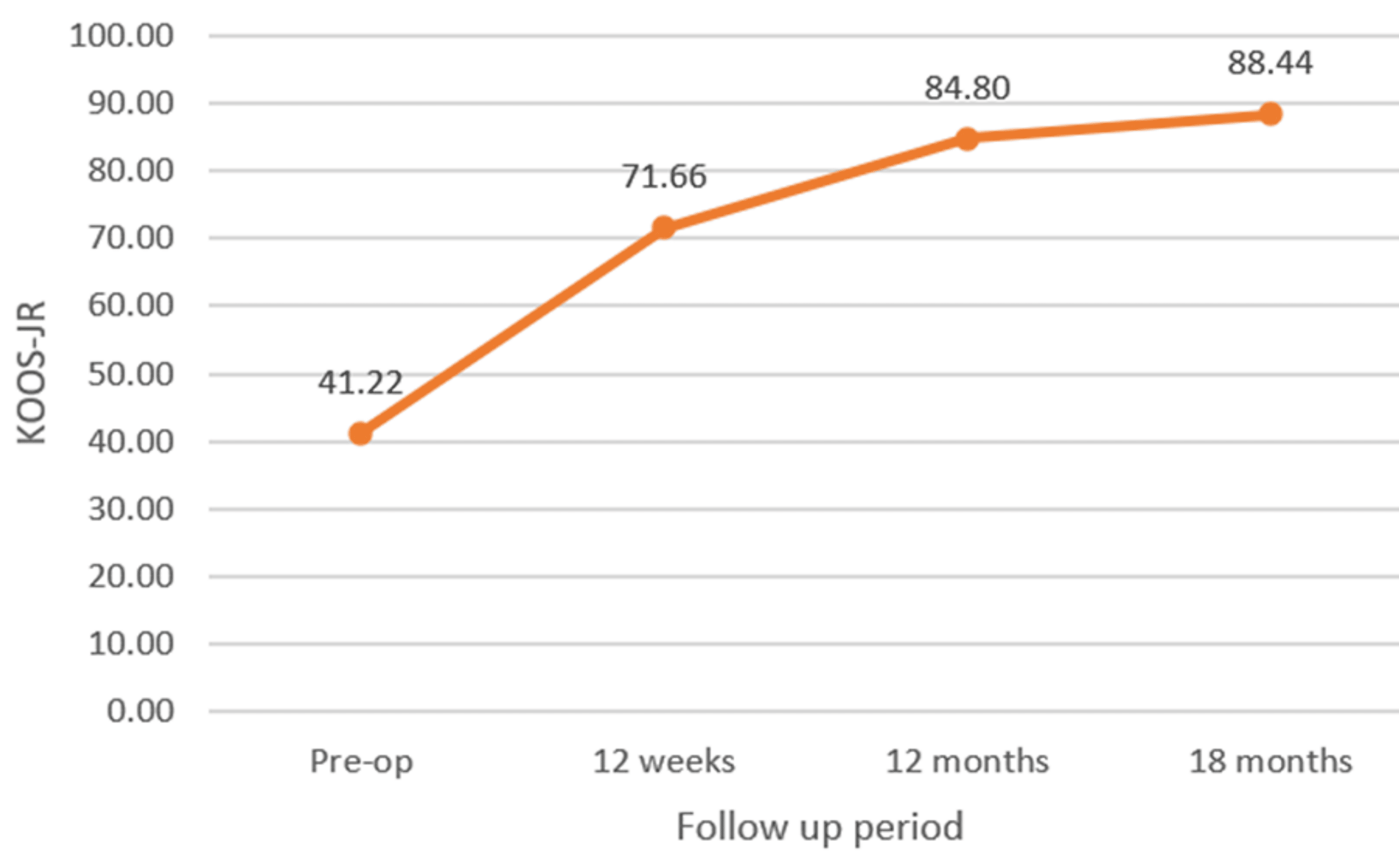
Line diagram depicting improving trend of KOOS-JR, suggesting improvement in the functional outcome at 18 months follow-up. KOOS-JR: Knee Injury and Osteoarthritis Outcome Score for Joint Replacement.

The KOOS-JR score showed a significant improvement from 41.22 ± 7.6 preoperatively to 71.66 ± 6.59 at 12 weeks postoperatively and to 84.8 ± 7.73 at 12 months and to 88.44 ± 8.23 at 18 months (p-value < 0.05) (Figure 8).

Functional outcomes in severe varus deformity (mean varus = 15.74° ± 5.17°) were found to be comparable to mild varus deformity (mean varus = 6.96° ± 2.03°) at three, 12, and 18 months with no statistical difference among the two (Table 3).

**Table 3:** Comparison of functional outcomes in mild versus severe varus groups. * P-value > 0.05 using Student's t-test, indicating no statistically significant difference. VAS-P: Visual Analogue Score for Pain; KOOS-JR: Knee Injury and Osteoarthritis Outcome Score for Joint Replacement.

	Mild varus, n = 26 (mean = 6.96° ± 2.03°)	Severe varus, n = 53 (mean = 15.74° ± 5.17°)	p-value
Preoperative VAS-P	7.45 ± 0.77	8.1 ± 0.75	<0.001
VAS-P at 3 months	3.78 ± 0.99	3.86 ± 1.3	0.753*
VAS-P at 12 months	2.11 ± 0.12	2.31 ± 1.56	0.569*
VAS-P at 18 months	1.87 ± 0.12	2.05 ± 1.57	0.593*
Preoperative KOOS-JR	42.12 ± 7.8	40.87 ± 7.59	0.498*
KOOS-JR at 3 months	73.08 ± 5.28	70.89 ± 7.22	0.173*
KOOS-JR at 12 months	85.85 ± 4.48	84.25 ± 9.07	0.399*
KOOS-JR at 18 months	89.73 ± 4.69	87.62 ± 9.63	0.295*

Out of 82 TKA, there were 15 cases (18.29%) who complained of anterior knee pain (AKP), and one patient (1.22%) had superficial fat necrosis, which did not require any intervention. No deep infection, wound dehiscence, or arthrofibrosis was reported.

The amount of bone resection done from the tibia and femur from each condyle was noted and is depicted in Table 4. There was a significant difference in bone resection from the medial tibial and medial distal femoral condyle (p-value < 0.05) with less bone resected in the severe varus group. The amount of bone resected from the lateral tibial, distal lateral femoral, and posterior femoral condyles was comparable among the two groups (p-value > 0.05). The minimum polyethylene insert was used, i.e., size 9 mm for all cases except for three cases (3.66%) where the next available size (11 mm) was used.

**Table 4:** Amount of bone resected from tibial and femoral condyles in our study. * P-value < 0.05 using Student's t-test, indicating a statistically significant difference. SD: standard deviation.

Site	Overall, mean ± SD (range), mm	Mild varus group, mean ± SD (range), mm	Severe varus group, mean ± SD (range), mm	p-value
Medial tibial condyle	1.15 ± 1.66 (-6 to 5)	1.9 ± 1.35 (0 to 5)	0.75 ± 1.7 (-6 to 4)	0.003*
Lateral tibial condyle	4.54 ± 1.52 (0 to 7.5)	4.44 ± 1.39 (1 to 7)	4.66 ± 1.5 (1.5 to 7.5)	0.536
Distal femoral medial condyle	5.3 ± 2.04 (1 to 10)	6.4 ± 1.85 (2 to 10)	4.7 ± 1.93 (1 to 10)	<0.001*
Distal femoral lateral condyle	3.53 ± 2.06 (0 to 9.5)	3.88 ± 2.17 (0.5 to 8.5)	3.53 ± 1.94 (0 to 9.5)	0.463
Posterior femoral medial condyle	8.48 ± 1.38 (5 to 11.5)	8.62 ± 1.53 (6 to 11.5)	8.4 ± 1.34 (5 to 11.5)	0.516
Posterior femoral lateral condyle	4.88 ± 1.53 (2 to 8)	5.15 ± 1.33 (0 to 5)	4.73 ± 1.64 (2 to 8)	0.252

## Discussion

This study reports the functional outcome of robotic TKA (RTKA) in cases with severe varus deformities in the Indian population. In our study, the mean preoperative HKA was 167.15° (range = 177° to 148°). The postoperative LA was achieved from HKA 174° to 180° with a mean HKA of 175.78° ± 1.35°. A significant improvement in the VAS-P from 7.87 to 1.96 and KOOS-JR scores from 41.22 to 88.44 was observed at 18 months follow-up. The VAS-P and KOOS-JR scores in severe deformities were comparable to those in milder deformities (p-value < 0.05).

The robotic system provides intraoperative real-time feedback and helps the operating surgeon achieve the desired alignment, implant position, and perfectly balanced gaps. In cases with severe deformities, this feedback is very important and helps in attaining optimal outcomes. The use of robotic systems for severe coronal plane deformities has been sparsely reported in the literature. Marchand et al. [13] successfully corrected a total of 129 patients with a mean varus of 10° (range = 7° to 18°) to postoperative varus 0°-7° using the MAKO robotic system. They, however, did not report the clinical outcomes in their study. Rossi et al. [14] showed a significant improvement in function using the robotic system in cases with a minimum 15° varus and 10° valgus deformity. However, they used a more constrained implant, i.e., constrained condylar knee (CCK) for varus knees and posterior stabilized knee (PS) for valgus knees, and had a short follow-up period of six months. Our study differed from this as all of our patients received unconstrained implants. A total of 77 knees (93.9%) received CR implants and five (6.1%) received CS implants.

There were a couple of other studies that have reported outcomes of RTKA in severe deformities [15,16]; however, the deformities in these studies were milder (less than 10° deviation of HKA) when compared to our study (HKA range = 177° to 148°) or they had a very short follow-up of 12 weeks. Table 5 provides details of studies that have reported the use of robotic systems in severe coronal plane deformities.

**Table 5:** Various studies reporting severe deformity correction using a robotic system. aMA: adjusted mechanical alignment; CCK: constrained condylar knee; CR: cruciate retaining; CS: cruciate substituting; FA: functional alignment; FJS: Forgotten Joint Score; HSS: Hospital for Special Surgery knee score; KOOS-JR - Knee Injury and Osteoarthritis Outcome Score for Joint Replacement; KSS: Knee Society Score; LA: limb alignment; OKS: Oxford Knee Score; PS: posterior stabilized knee; VAS-P: Visual Analogue Score for Pain; WOMAC: Western Ontario and McMaster Universities Arthritis Index.

Study	Number of knees	Preoperative LA (°)	Postoperative LA (°)	Clinical outcome	Implant used
Marchand et al. [13]	n = 129	Varus: 7 to 18 (mean = 10)	0 to 7	Not reported	Triathlon, CR
	n = 7	Valgus: 7 to 12 (mean = 9)	0 to 3	Not reported	
Rossi et al. [14]	n = 20	Minimum varus = 15	-	Improved KSS, HSS, OKS, and WOMAC score	CCK
	n = 10	Minimum valgus = 10	-		PS
Jeffrey et al. [10]	n = 64 (FA)	15 to -11	0 to 8	FA showed better FJS, KSS, and OKS scores	60 CR, 4 PS, all patella resurfaced
	n = 64 (aMA)	15 to -7	0 to 7		58 CR, 6 PS, all patella resurfaced
Our study	n = 79	Varus: 3 to 32 (mean = 12.85)	0 to 6	Improvement in VAS-P and KOOS-JR scores	Triathlon, CR - 77, CS - 5, patelloplasty and denervation
	n = 3	Valgus: 3 to 10	0 to 4		

Several alignment options are available and can be used with robotic systems [17]. The use of FA with robotic assistance for the performance of TKA is gaining popularity. It has been noted that this alignment strategy has most consistently provided a functional and stable knee without the need for ligamentous releases in most cases [18]. Van de Graaf et al. [19] analyzed MA, KA, and FA. They found that FA provided the highest proportion of balanced knee (96.5%) in all four gap measurements as compared to MA (54.7%) and KA (66.4%). Similar findings were reported by Clark et al. who observed a 97% balance with FA without soft tissue release [20]. In their study, Jeffrey et al. [10] reported that FA gives a better clinical outcome in short-term follow-up (12-15 months). The preoperative coronal deformity ranged from 0° to 15° varus and 0° to 11° valgus. In contrast, our study had 23 knees (29.11%) with more than 15° varus, which was corrected to 0° to 6° varus using the FA strategy. A total of 73 knees (89.02%) of cases achieved the MCID for VAS-P and 77 knees (93.9%) achieved the MCID for KOOS-JR in our study.

Having said that, nine (10.98%) of our cases still had pain as the main residual symptom. Fifteen of our cases (18.29%) had AKP despite good patellar tracking and no overstuffing of the patellofemoral joint. Motififard et al. [21] reported that neurectomy provided significant postoperative difference only up to three weeks after surgery in TKA without patella resurfacing. Another review article reported a global incidence of AKP of 11.3% after patelloplasty [22]. They concluded that it was a superior option when compared to denervation and osteophyte removal alone. We combined patelloplasty with neurectomy for all our cases and still 15 cases (18.29%) had AKP at the final follow-up.

Analysis of the bone resection pattern in varus knees was performed. We found that the mean bone resected from the medial tibial condyle was 1.13 ± 1.68 mm, ranging from -6 mm to 5 mm. The negative sign here denotes a bone defect of that many millimeters after final cuts. It was filled up using bone cement in all cases. There were 16 such cases (19.51%) in which no bone was resected at all from the medial tibial condyle. Also, the tibial component was placed at a mean varus of 2.54° ± 0.83°. The overall effect is bone conservation on the lateral tibial condyle. The amount of bone resected from the lateral tibial, distal lateral femoral, and posterior femoral condyles was comparable among the two groups (p-value > 0.05) (Table 4).

Van de Graaf et al. [19], in their comparative study, reported that MA resects minimum bone from the medial tibia plateau. However, this does not imply that MA is the most bone-conserving as the amount of bone resected from the lateral tibial plateau is much higher. Mannan et al. [23] found that mean resection from the unaffected tibial plateau was 7.6 mm with MA and > 10 mm resection was performed in 17% of the knees amounting to a significant amount of bone loss.

The FA workflow favors femoral component placement in less valgus when compared to MA [10]. This could be validated in our study. The mean lateral distal femoral condyle bone cut was 3.65 ± 2.01 mm, as compared to 5.26 ± 2.06 mm on the medial distal femur condyle. The femoral component was placed at a mean varus of 0.13° ± 0.35° (range = 1° varus to 1° valgus). The measured resection takes away 9 mm of bone during the distal femur cut. FA proves to be more bone preserving for distal femur bone and a similar finding was reported by Van de Graaf et al. [19].

All the cases were performed by a single senior experienced surgeon at a single center. This allowed us to use a single standardized surgical technique and alignment strategy with a single implant (Stryker Triathlon) with the MAKO robotic assistance. This was one of the main strengths of this study. Also, we additionally calculated MCID for both VAS-P and KOOS-JR scores as a statistically significant difference may not always demonstrate clinical usefulness.

There were a few limitations. There was no comparative group in our study; however, our primary aim was to report the clinical outcomes with severe varus deformities in TKA using robotic arm assistance and not to compare with other methodologies. There were only three cases with valgus deformity and therefore no meaningful conclusion could be drawn for valgus knees. A small sample size was another limitation. A longer follow-up would have added more value to our findings.

## Conclusions

The use of functional alignment with robotic assistance to correct severe varus deformities in TKA showed a significant reduction in VAS-P scores and an improvement in KOOS-JR scores. This approach helps in attaining optimal outcomes in these patients with severe varus. The functional outcomes were comparable in severe (mean varus = 15.74⁰ ± 5.17) and mild varus (mean varus = 6.96⁰ ± 2.03⁰) deformities. Functional alignment also helps in conserving the bone from the lateral tibial condyle and distal femur condyles even in severe varus deformity cases by minimizing the amount of resected bone.
